# Engineering a bzd cassette for the anaerobic bioconversion of aromatic compounds

**DOI:** 10.1111/1751-7915.12746

**Published:** 2017-07-24

**Authors:** María Teresa Zamarro, María J. L. Barragán, Manuel Carmona, José Luis García, Eduardo Díaz

**Affiliations:** ^1^ Centro de Investigaciones Biológicas CSIC C/Ramiro de Maeztu, 9 28040 Madrid Spain; ^2^ Present address: U.S. Food and Drug Administration Center for Drug Evaluation and Research (CDER) 10903 New Hampshire Avenue Silver Spring MD 20993 USA

## Abstract

Microorganisms able to degrade aromatic contaminants constitute potential valuable biocatalysts to deal with a significant reusable carbon fraction suitable for eco‐efficient valorization processes. Metabolic engineering of anaerobic pathways for degradation and recycling of aromatic compounds is an almost unexplored field. In this work, we present the construction of a functional bzd cassette encoding the benzoyl‐CoA central pathway for the anaerobic degradation of benzoate. The bzd cassette has been used to expand the ability of some denitrifying bacteria to use benzoate as sole carbon source under anaerobic conditions, and it paves the way for future pathway engineering of efficient anaerobic biodegraders of aromatic compounds whose degradation generates benzoyl‐CoA as central intermediate. Moreover, a recombinant *Azoarcus* sp. CIB strain harbouring the bzd cassette was shown to behave as a valuable biocatalyst for anaerobic toluene valorization towards the synthesis of poly‐3‐hydroxybutyrate (PHB), a biodegradable and biocompatible polyester of increasing biotechnological interest as a sustainable alternative to classical oil‐derived polymers.

## Introduction

Aromatic compounds are the second most abundant class of organic compounds in nature after carbohydrates. Due to the thermodynamic stability of the aromatic ring, aromatic compounds are difficult to degrade and they tend to persist in the environment for long periods of time. Many of these compounds are toxic and/or carcinogenic thus representing major persistent environmental pollutants. Therefore, removal of aromatic compounds is very important both for a balanced global carbon budget and to protect wildlife and human health. Some specialized microorganisms (bacteria, archaea and fungi) have adapted to use aromatic compounds as sole carbon and energy source (mineralization) or, at least, partially degrade these molecules to less‐toxic and persistent compounds (Carmona *et al*., 2009; Fuchs *et al*., 2011). These microorganisms constitute, thus, potential valuable biocatalysts to deal with a significant reusable carbon fraction suitable for eco‐efficient valorization processes within the framework of a sustainable knowledge‐based bio‐economy.

There are two major strategies to degrade aromatic compounds depending on the presence or absence of oxygen. In the aerobic catabolism of aromatic compounds, oxygen is not only the final electron acceptor but also a cosubstrate for two key processes, i.e. the hydroxylation and oxygenolytic ring cleavage of the aromatic ring, carried out by oxygenases. In the absence of oxygen (anaerobic catabolism), the aromatic ring is de‐aromatized by reductive reactions (Carmona *et al*., 2009; Fuchs *et al*., 2011; Boll *et al*., 2014; Rabus *et al*., 2016). A wide variety of bacteria, pathways and associated gene clusters responsible for the aerobic catabolism of aromatic compounds have been studied and characterized (Díaz *et al*., 2013). Moreover, genetic and metabolic engineering approaches have been applied to develop more efficient recombinant biocatalysts for the aerobic conversion of aromatic compounds to added value products, e.g. biopolymers, biofuels, commodity chemicals (Kosa and Ragauskas, 2012; Bugg and Rahmanpour, 2015; Wierckx *et al*., 2015; Beckham *et al*., 2016; Johnson *et al*., 2016). In contrast, the anaerobic degradation of aromatic compounds has been much less well‐studied than the aerobic degradation, especially regarding the genetic determinants that encode the anaerobic pathways, and there are only a few examples of recombinant anaerobic biodegraders (Coschigano *et al*., 1994; Darley *et al*., 2007; Zamarro *et al*., 2016). However, anaerobic processes may offer significant benefits compared to aerobic bioprocesses, e.g. higher yields, less heat and oxidative stress generation, reduced biomass production and lower mechanical energy input, as they do not require aeration, which can significantly reduce production costs (Cueto‐Rojas *et al*., 2015). Thus, metabolic engineering of anaerobic pathways for degradation and recycling of waste aromatic compounds is still an almost unexplored field of great biotechnological potential.

Similar to the very well‐known aerobic degradation strategies, the anaerobic degradation of aromatic compounds channels a wide variety of compounds into a few central intermediates through devoted peripheral degradation pathways (catabolic funnel). The different peripheral pathways converge into a few central pathways that carry out the reductive de‐aromatization and further conversion of the central intermediates to compounds of the central metabolism of the cell. Most monocyclic aromatic compounds are channelled and activated to benzoyl‐CoA. The catabolic genes encoding the benzoyl‐CoA central pathway enzymes are usually arranged in large chromosomal clusters that also contain the specific transcriptional regulators (Egland *et al*., 1997; Breese *et al*., 1998; López‐Barragán *et al*., 2004; Rabus *et al*., 2005, 2016; Wischgoll *et al*., 2005; Carmona *et al*., 2009; Holmes *et al*., 2012; Carlström *et al*., 2015; Hirakawa *et al*., 2015).

*Azoarcus* sp. CIB is a facultative anaerobic beta‐proteobacterium capable of degrading either aerobically and/or anaerobically (using nitrate as terminal electron acceptor) a wide range of aromatic compounds including some toxic hydrocarbons such as toluene (López‐Barragán *et al*., 2004; Martín‐Moldes *et al*., 2015; Zamarro *et al*., 2016). Several pathways involved in the anaerobic degradation of aromatic compounds, including the benzoyl‐CoA central pathway (*bzd* genes), have been characterized at the molecular level in strain CIB (López‐Barragán *et al*., 2004; Carmona *et al*., 2009; Juárez *et al*., 2013). In addition to this free‐living lifestyle, *Azoarcus* sp. CIB also shows an endophytic lifestyle (Fernández *et al*., 2014) and is able to resist some metals and metalloids, e.g. selenium oxyanions, producing nanoparticles of biotechnological interest (Fernández‐Llamosas *et al*., 2016). All these properties, together with the fact that the genome of strain CIB has been sequenced and annotated and different tools are available for its genetic manipulation (Martín‐Moldes *et al*., 2015), make *Azoarcus* sp. CIB a promising host for approaching metabolic engineering strategies to improve the anaerobic bioconversion of aromatic compounds.

In this work, we present the construction of a functional bzd cassette for anaerobic benzoate degradation and its application to the development of recombinant *Azoarcus* sp. CIB biocatalysts for toluene valorization towards the synthesis of poly‐3‐hydroxybutyrate (PHB), a biodegradable and biocompatible polyester of increasing biotechnological interest as a sustainable alternative to classical oil‐derived polymers (Rehm, 2010; Nikodinovic‐Runic *et al*., 2013; Madbouly *et al*., 2014).

## Results and discussion

### Construction of a functional bzd catabolic cassette for anaerobic degradation of benzoate

**Figure 1:**
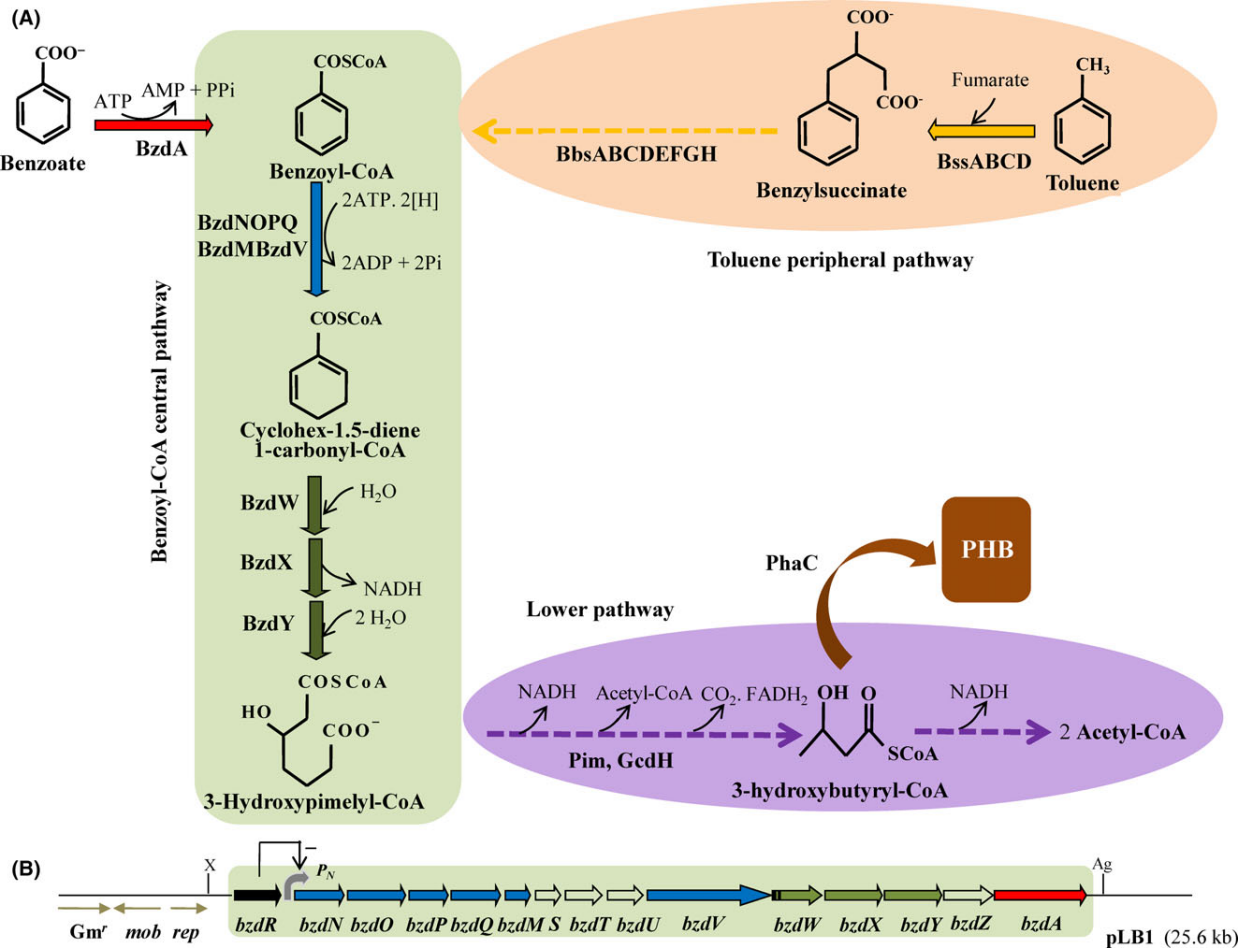
Scheme of the anaerobic metabolism of benzoate and toluene, and gene organization of the bzd cassette in *Azoarcus* sp. CIB. A. Scheme of peripheral pathway for the anaerobic conversion of toluene into benzoyl‐CoA (orange), the activation of benzoate to benzoyl‐CoA (red), the benzoyl‐CoA central pathway (green), the lower pathway (violet) and the polymerization of 3‐hydroxybutyryl‐CoA to PHB (brown). Discontinuous arrows indicate that more than one enzymatic step is involved. Enzyme abbreviations: BssABCD, benzylsuccinate synthase; Bbs, enzymes involved in the modified β‐oxidation of benzylsuccinate to benzoyl‐CoA; BzdA, benzoate‐CoA ligase; BzdNOPQ, benzoyl‐CoA reductase; BzdM, ferredoxin; BzdV, putative NADPH:ferredoxin oxidoreductase; BzdW, cyclohex‐1,5‐diene‐1‐carbonyl‐CoA hydratase; BzdX, 6‐hydroxycyclohex‐1‐ene‐1‐carbonyl‐CoA dehydrogenase; BzdY, 2‐ketocyclohexane‐1‐carbonyl‐CoA hydrolase; Pim, enzymes involved in β‐oxidation of dicarboxylic acids; GcdH, glutaryl‐CoA dehydrogenase; PhaC, PHB synthase. B. Schematic representation of the *bzd* genes for anaerobic benzoate degradation engineered as a mobile DNA cassette in plasmid pLB1. Genes are indicated in the same colour code than the corresponding enzymes in panel A; i.e., genes encoding enzymes involved in the initial activation, de‐aromatization and modified β‐oxidation are indicated in red, blue and green colour respectively. The *bzdR* gene encoding a transcriptional repressor of the catabolic *P*_*N*_ promoter is shown in black. X and Ag, XbaI and AgeI restriction sites flanking the bzd cassette respectively. The gentamicin resistance gene (Gm^r^), mobilization (*mob*) and replication (*rep*) functions are also indicated.

**Figure 2:**
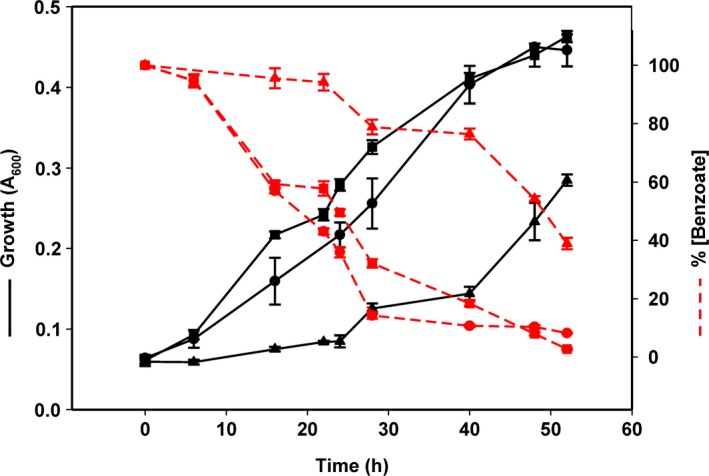
Anaerobic growth curves and benzoate consumption in different *Azoarcus* strains. *Azoarcus* sp. CIB (pIZ1016) (circles), *Azoarcus* sp. CIBd*bzdN* (pLB1) (squares) and *Azoarcus communis* (pLB1) (triangles) were grown anaerobically at 30°C in minimal MC medium containing 3 mM benzoate and 10 mM nitrate as sole donor and electron acceptors respectively, as previously detailed (López‐Barragán *et al*., 2004). Gentamicin (7.5 μg ml^−1^) was added to the medium to assure plasmid maintenance. Bacterial growth (black lines) was monitored by measuring the absorbance at 600 nm (*A*_600_). The concentration of benzoate in the culture medium (red lines) was monitored spectrophotometrically at 273 nm (López‐Barragán et al., 2004) and is indicated as a percentage of the initial concentration. Values are the mean of three different experiments. Error bars indicate standard deviation.

As indicated above, the benzoyl‐CoA central pathway involved in the anaerobic degradation of benzoate and many other aromatic compounds whose peripheral pathways converge into benzoyl‐CoA has been previously characterized in the denitrifying *Azoarcus* sp. CIB strain (López‐Barragán *et al*., 2004; Carmona *et al*., 2009). Benzoate becomes initially activated to benzoyl‐CoA by the benzoate‐CoA ligase (BzdA) and is then de‐aromatized by the action of a benzoyl‐CoA reductase (BzdNOPQ), the only oxygen‐sensitive enzyme within the benzoyl‐CoA pathway, that uses a low‐potential ferredoxin (BzdM) as electron donor and generates cyclohexa‐1,5‐diene‐1‐carbonyl‐CoA (1,5‐dienoyl‐CoA) (Fig. 1A). Further degradation of 1,5‐dienoyl‐CoA resembles a modified β‐oxidation pathway with addition of water to a double bond (BzdW dienoyl‐CoA hydratase), a dehydrogenation reaction (BzdX hydroxyacyl‐CoA dehydrogenase) and hydrolytic ring fission (BzdY oxoacyl‐CoA hydrolase), generating finally 3‐hydroxy‐pimelyl‐CoA that feeds into a lower pathway (Fig. 1A). The *bzd* genes encoding the bzd pathway enzymes are clustered together in a large operon driven by the *P*_*N*_ promoter (Fig. 1B). The specific transcriptional regulation of the *bzd* operon is conducted by the BzdR repressor that is encoded immediately upstream of the catabolic operon (Fig. 1B). Induction of the *bzd* genes requires the binding of the effector molecule, benzoyl‐CoA, to the BzdR repressor (Durante‐Rodríguez *et al*., 2010). The *bzdNOPQMSTUVWXYZA* catabolic genes and the cognate *bzdR* regulatory gene have been engineered as a 19.6 kb DNA cassette into a broad‐host range vector, giving rise to plasmid pLB1 (Fig. 1B). To construct the bzd cassette, the right end of the *bzd* cluster (genes *bzdXYZA*) from plasmid pECOR8 (Table 1) was first cloned as an EcoRI fragment into the EcoRI‐digested broad‐host range pIZ1016* vector, giving rise to plasmid pIZECO (Table 1). Then, the left end of the *bzd* cluster (genes *bzdRbzdNOPQMSTUVWX*) was cloned as a XbaI/NcoI‐double digested fragment from the recombinant λBzd1 phage (López‐Barragán *et al*., 2004) into the XbaI/NcoI‐double digested pIZECO plasmid, giving rise to plasmid pLB1 (25.6 Kb) (Table 1). To check whether the bzd cassette was functional, it was transferred to a mutant strain, *Azoarcus* sp. CIBd*bzdN,* unable to use benzoate anaerobically because it contains a disruption insertion in the first gene of the *bzd* catabolic operon with avoids the expression of the rest of *bzd* genes (Table 1) (López‐Barragán *et al*., 2004). Plasmid pLB1 was transferred by biparental filter mating from *E. coli* S17‐1λ*pir* (donor strain) to *Azoarcus* sp. CIBd*bzdN* (recipient strain) as previously described (López‐Barragán *et al*., 2004). Exconjugants harbouring the pLB1 plasmid, *Azoarcus* sp. CIBd*bzdN* (pLB1) (Table 1), were isolated aerobically on gentamicin (7.5 μg ml^−1^)‐containing MC medium with 10 mM glutarate as sole carbon source for counterselection of donor cells. The presence of plasmid pLB1 in *Azoarcus* sp. CIBd*bzdN* cells restored their anaerobic growth on benzoate and caused the consumption of this carbon source, as in the case of the wild‐type CIB strain containing plasmid pIZ1016 as control (Fig. 2). This result strongly suggested that the recombinant bzd cassette in plasmid pLB1 was functional. To confirm this, plasmid pLB1 was transferred to a closely related species, *Azoarcus communis* SWub3 (Table 1), that is an endophyte unable to degrade aromatic compounds under anaerobic conditions (Reinhold‐Hurek *et al*., 1993) but that can use aliphatic dicarboxylic acids, e.g. glutarate, that feed to the lower benzoyl‐CoA pathway (Fig. 1A). Remarkably, the recombinant *A. communis* SWub3 (pLB1) strain was able to grow anaerobically using benzoate as sole carbon and energy source (doubling time of about 15 h), confirming that the bzd cassette was functional in heterologous hosts and conferred the ability to degrade benzoate in anoxic conditions (Fig. 2).

**Table 1:** Bacteria and plasmids used in this study

Strain or plasmid	Relevant genotype and main characteristics	Reference or source
*E. coli* strains		
DH5α	*endA1 hsdR17 supE44 this‐1 recA1 gyrA(Nar* ^*r*^ *) relA1 Δ/argF‐lac) U169 depRФ80dlacd(lacZ)M15*	Sambrook and Russell, (2001)
S17‐1λpir	Tp^r^ Sm^r^ *recA thi hsdRM* ^+^ RP4׃׃2.Tc׃׃Mu׃׃Km Tn7 *λpir* phage lysogen	de Lorenzo and Timmis, (1994)
*Azoarcus* strains		
*Azoarcus* sp. CIB	Wild‐type strain	López‐Barragán *et al*., (2004)
*Azoarcus* sp. CIBd*bzdN*	Km^r^ *, Azoarcus* sp. CIB with a disruption in the *bzdN* gene	López‐Barragán *et al*., (2004)
*Azoarcus communis* SWub3	Wild‐ type strain (LMG22127)	Reinhold‐Hurek *et al*., (1993)
*Azoarcus* sp. CIB (pLB1)	CIB strain containing plasmid pLB1	This work
*Azoarcus* sp. CIBd*bzdN* (pLB1)	CIBd*bzdN* strain containing plasmid pLB1	This work
*A. communis* SWub3 (pLB1)	SWub3 strain containing plasmid pLB1	This work
Plasmids		
pECOR8	Ap^r^, pUC19 harbouring a 5.4 Kb EcoRI DNA fragment from *Azoarcus* sp. CIB that contains the right end of the *bzd* cluster	López‐Barragán *et al*., (2004)
pIZ1016	Gm^r^, pBBR1MCS‐5 broad‐host range cloning vector	Moreno‐Ruiz *et al*., (2003)
pIZ1016*	Gm^r^, pIZ1016 derivative without the NcoI restriction site in the polylinker	This work
pIZECO	Gm^r^, 11.4 Kb‐pIZ1016* derivative harbouring the right end of the *bzd* cluster	This work
pLB1	Gm^r^, 25.6 Kb‐pIZ1016* derivative harbouring the complete *bzd* cassette	This work

As far as we know, this is the first report on the construction of a DNA cassette encoding a transferable benzoyl‐CoA central pathway that allows the expansion of the catabolic potential of certain facultative anaerobes towards the use of aromatic compounds under anaerobic conditions. Moreover, this result reinforces the recent thought that questions the previous idea that the *Azoarcus* genus comprises bacteria that fit into one of two major eco‐physiological groups, i.e. either the free‐living anaerobic biodegraders of aromatic compounds or the obligate endophytes unable to degrade aromatics under anaerobic conditions (Fernández *et al*., 2014). Thus, here we show that a member of the subgroup of *Azoarcus* strains that are obligate endophytes unable to degrade aromatics under anaerobic conditions can evolve towards the use of these carbon sources when acquiring the cognate genetic determinants.

### *Azoarcus* sp. CIB accumulates PHB when grown anaerobically in toluene

**Figure 3:**
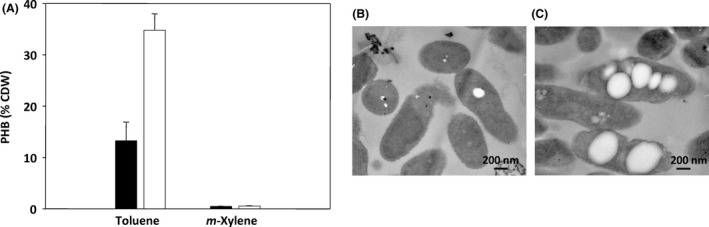
Analysis of *Azoarcus* sp. CIB cells that contain plasmid pIZ1016 (control) or the pLB1 plasmid grown anaerobically on aromatic hydrocarbons. A. PHB monomer content of *Azoarcus* sp. CIB (pIZ1016) (black bars) and *Azoarcus* sp. CIB (pLB1) (white bars) cells at mid‐exponential phase of anaerobic growth at 30°C in minimal MC medium with 400 mM toluene or 275 mM *m*‐xylene, supplied in 2,2,4,4,6,8,8‐heptamethylnonane as an inert carrier phase, and 10 mM nitrate. PHB production was quantified by GC‐MS of the methanolysed polyester and shown as the percentage of PHB monomer with respect to the total cell dry weight (CDW). Error bars represent standard derivation found in four different experiments. Methanolysis procedure was carried out by suspending 5–10 mg of lyophilized cells in 0.5 ml of chloroform and 2 ml of methanol containing 15% sulfuric acid and 0.5 mg ml^−1^ of 3‐methylbenzoic acid (internal standard), and then incubated at 80°C for 7 h. After cooling, 1 ml of de‐mineralized water and 1 ml of chloroform were added and the organic phase containing the resulting methyl esters of monomers was analysed by GC‐MS (de Eugenio *et al*., 2010). An Agilent series 7890A coupled with 5975C MS detector (EI, 70 eV) and a split–splitless injector were used for analysis. An aliquot (1μl) of organic phase was injected into the gas chromatograph at a split ratio 1:20. Separation of compounds was achieved using an HP‐5 MS capillary column (5% phenyl‐95% methyl siloxane, 30 m × 0.25 mm film thickness). Helium was used as carrier gas at a flow rate of 1 ml min^−1^. The injector and transfer line temperature were set at 275 and 300°C respectively. The oven temperature programme was as follows: initial temperature 80°C for 2 min, then from 80°C up 150°C at a rate of 5°C min^−1^ and held for 1 min. The mass spectra were recorded in full scan mode (m/z 40–550). 3‐hydroxybutyric acid methyl ester was resolved using selected ion monitoring mode (SIM). B and C. Representative view of *Azoarcus* sp. CIB (pIZ1016) (B) and *Azoarcus* sp. CIB (pLB1) (C) cells grown anaerobically on toluene. Cells were harvested, washed twice in PBS and fixed in 5% (w/v) glutaraldehyde. Afterwards, cells were suspended in 2.5% (w/v) OsO_4_ for 1 h, gradually dehydrated in ethanol solutions [30%, 50%, 70%, 90% and 100% (v/v); 30 min each) and propylene oxide (1 h), and embedded in Epon 812 resin. Ultrathin sections were cut with a microtome using a Diatome diamond knife. The sections were picked up with 400‐mesh cupper grids coated with a layer of carbon and observed using a Jeol‐1230 transmission electron microscope. PHB granules can be observed as white spheres inside the cells.

It has been reported that some anaerobic biodegraders of aromatic hydrocarbons, such as the bacterium *Aromatoleum aromaticum* EbN1, are able to accumulate poly(3‐hydroxybutyrate) (PHB) up to 5.2% of the cell dry weight (CDW) during anaerobic growth on toluene using nitrate as final electron acceptor (Trautwein *et al*., 2008). PHB formation was predicted to contribute at two different levels, (i) enhancing consumption of surplus reducing equivalents generated during the anaerobic catabolism of aromatic hydrocarbons, and (ii) alleviating the cytotoxic effect of aromatic hydrocarbons by trapping them into the hydrophobic PHB granules (Trautwein *et al*., 2008; Rabus *et al*., 2014). To check whether *Azoarcus* sp. CIB was also able to accumulate PHB when grown anaerobically on toluene, PHB monomer composition and cellular PHB content of lyophilized cells were determined by gas chromatography‐tandem mass spectrometry (GC‐MS) of the methanolysed polyester as previously described (de Eugenio *et al*., 2010). The chromatographic profile obtained by GC‐MS of the products released showed a single peak corresponding to the 3‐hydroxy‐butyrate monomer of the PHB polymer. Figure 3A shows that *Azoarcus* sp. CIB cells grown anaerobically on toluene were able to accumulate PHB at a level of 13% CDW. Moreover, granules of PHB were clearly observed in *Azoarcus* sp. CIB cells by transmission electron microscopy (Fig. 3B).

Taken together, these results show that *Azoarcus* sp. CIB is able to degrade toluene anaerobically and eventually reroute some of the metabolic flux towards the synthesis of a valuable biopolymer such as PHB. According to the known peripheral pathway for anaerobic toluene degradation in denitrifying bacteria, this aromatic hydrocarbon becomes activated to benzylsuccinate by the *bssABCD*‐encoded benzylsuccinate synthase enzyme, and then the later is converted to benzoyl‐CoA via a modified β‐oxidation pathway encoded by the *bbsABCDEFJGH* genes (Fig. 1A) (Heider *et al*., 2016; Zamarro *et al*., 2016). Benzoyl‐CoA is then converted to 3‐hydroxypimelyl‐CoA via the upper benzoyl‐CoA central pathway (Fig. 1A), and the later is further metabolized to the central metabolism by a dicarboxylic acid β‐oxidation pathway (lower benzoyl‐CoA pathway) (Fig. 1A) (López‐Barragán *et al*., 2004; Carmona *et al*., 2009). As one of the final metabolites of the lower benzoyl‐CoA pathway, i.e. 3‐hydroxybutyryl‐CoA, is the monomer used by the PHB synthase to produce the PHB polymer (Fig. 1A) (Rehm, 2010), the anaerobic metabolism of toluene might stimulate PHB production by increasing the 3‐hydroxybutyryl‐CoA levels in the cell. Therefore, it is tempting to speculate that increasing the flux through the benzoyl‐CoA pathway could further enhance the anaerobic conversion of toluene into PHB. In *Azoarcus* sp. CIB, the *bss‐bbs* genes are clustered together within an integrative and conjugative element ICE_*XTD*_ that enhances their copy dosage in the cell (Martín‐Moldes *et al*., 2015; Zamarro *et al*., 2016), thus likely favouring the initial activation of toluene by benzylsuccinate synthase, a reaction that was shown to be a rate‐limiting step in toluene catabolism. As several gene clusters have been predicted to encode dicarboxylic acid β‐oxidation pathways in strain CIB (Martín‐Moldes *et al*., 2015), it appears that the single gene dosage *bzd* genes could represent a bottleneck when trying to enhance the metabolic flux through the benzoyl‐CoA pathway for achieving a higher conversion of toluene into PHB. Thus, the use of the pLB1 multicopy plasmid could be a rational strategy to increase the *bzd* gene dosage and, eventually, enhance the anaerobic production of PHB in *Azoarcus* sp. CIB.

### The bzd cassette enhances PHB accumulation in a recombinant *Azoarcus* sp. CIB (pLB1) strain

**Figure 4:**
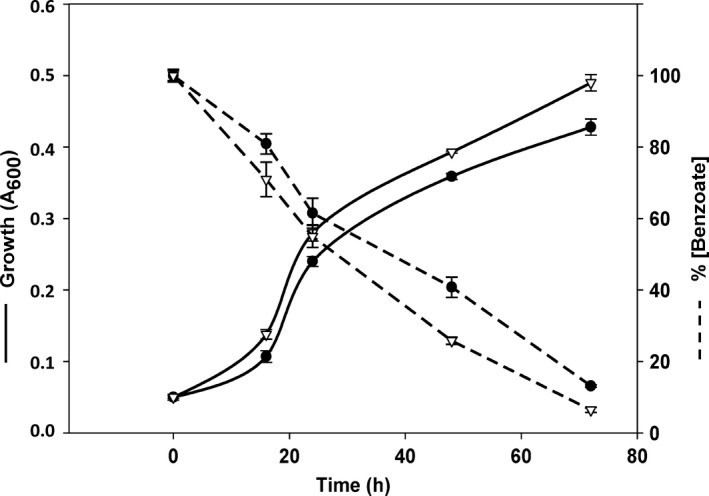
Growth and benzoate consumption of *Azoarcus* sp. CIB harbouring plasmid pIZ1016 (circles) or the pLB1 plasmid (triangles). Bacteria were cultivated anaerobically at 30°C in minimal MC medium containing 3 mM benzoate and 10 mM nitrate. Bacterial growth, monitored by measuring the absorbance at 600 nm (*A*_600_), is indicated with a continuous line. The concentration of benzoate in the culture medium was monitored spectrophotometrically at 273 nm, and the percentage of benzoate remaining in the culture medium is indicated with a dashed line. Values are the mean of three different experiments. Error bars indicate standard deviation.

To check whether the expression of the *bzd* genes in the multicopy plasmid pLB1 could lead to a higher anaerobic benzoate metabolism, growth and benzoate consumption in *Azoarcus* sp. CIB (pLB1) cells were compared with those in the wild‐type *Azoarcus* sp. CIB cells. As shown in Fig. 4, a slightly increase in growth and benzoate removal was observed in cells containing the pLB1 plasmid, suggesting that increasing the *bzd* gene dosage enhances the anaerobic degradation of benzoate in *Azoarcus* sp. CIB. Then, we monitored the accumulation of PHB in *Azoarcus* sp. CIB (pLB1) cells grown anaerobically on 400 mM toluene supplied in 2,2,4,4,6,8,8‐heptamethylnonane carrier phase. PHB production was quantified from cells harvested at mid‐exponential phase, as previously done with the wild‐type CIB strain. Interestingly, the bzd cassette caused accumulation of PHB up to 35% CDW in *Azoarcus* sp. CIB (pLB1) (Fig. 3A). Thus, these results show that when recombinant *Azoarcus* sp. CIB (pLB1) cells grow anaerobically on toluene, the bzd cassette enhances 2.7‐fold the bio‐production of PHB with respect to that observed in wild‐type cells. The higher conversion of toluene into PHB was also confirmed by transmission electron microscopy, which revealed an increase in number and size of PHB granules in recombinant cells (Fig. 3C) with respect to wild‐type cells (Fig. 3B). The level of PHB accumulation from the toxic hydrocarbon toluene was the highest described so far under anaerobic conditions, and it was even higher than that reported for the aerobic bioconversion of aromatic hydrocarbons to medium‐chain‐length polyhydroxyalkanoates (Ward *et al*., 2005; Nikodinovic *et al*., 2008; Ni *et al*., 2010; Narancic *et al*., 2012) or to PHB (Keum *et al*., 2008; Hori *et al*., 2009). As toluene is a major contaminant of high‐volume waste streams at places where it is produced or used (e.g. petrochemical industry, solvents and painting markets, biopolymers (e.g. poly(ethylene terephthalate), polyurethanes), its valorization becomes a sustainable strategy for the recycling industry (Wierckx *et al*., 2015).

*Azoarcus* sp. CIB is also able to grow anaerobically on *m*‐xylene as sole carbon and energy source (Juárez *et al*., 2013; Zamarro *et al*., 2016). Although the peripheral pathway for anaerobic *m*‐xylene degradation is likely encoded by the same *bss‐bbs* genes responsible for the toluene peripheral pathway, the central intermediate of *m*‐xylene degradation is 3‐methylbenzoyl‐CoA rather than benzoyl‐CoA (as in the case of toluene) (Juárez *et al*., 2013; Zamarro *et al*., 2016). The 3‐methylbenzoyl‐CoA is further degraded via a specific mbd anaerobic central pathway, which is different to the common bzd pathway, and a lower pathway that does not generate 3‐hydroxybutyryl‐CoA (Juárez *et al*., 2013; Zamarro *et al*., 2016). Thus, anaerobic *m*‐xylene degradation might not generate as much PHB as the anaerobic catabolism of toluene. In fact, when *Azoarcus* sp. CIB cells were grown in 275 mM *m*‐xylene, supplied in 2,2,4,4,6,8,8‐heptamethylnonane carrier phase, no significant PHB accumulation was detected (< 1% CDW) (Fig. 3A). As expected, no further increase of PHB accumulation was observed in *Azoarcus* sp. CIB (pLB1) cells grown on *m*‐xylene (Fig. 3A). Therefore, these results strongly suggest that the observed effect of the multicopy bzd cassette enhancing PHB production from toluene might be due to an enhanced metabolic flux through the anaerobic benzoyl‐CoA pathway.

## Conclusion

This work is a proof of concept of the potential of metabolic engineering for the anaerobic bioconversion of aromatic compounds towards the production of valuable products. We have engineered the first broad‐host range metabolic bzd cassette for the anaerobic degradation of benzoate. The bzd cassette has been used to expand the ability of some denitrifying bacteria to use benzoate as sole carbon source under anaerobic conditions, and it paves the way for future pathway engineering of efficient anaerobic biodegraders of aromatic compounds. Moreover, we have developed a recombinant *Azoarcus* sp. CIB strain harbouring the bzd cassette as a valuable environmentally friendly strategy for the efficient anaerobic conversion of some petrochemical waste into added value products, such as bioplastics, relevant to the circular economy.

## Conflict of Interest

The authors declare no conflict of interest.
